# Nanoengineering facilitating the target mission: targeted extracellular vesicles delivery systems design

**DOI:** 10.1186/s12951-022-01638-9

**Published:** 2022-09-29

**Authors:** Haoyue Song, Xiaohang Chen, Yujia Hao, Jia Wang, Qingpeng Xie, Xing Wang

**Affiliations:** 1 Shanxi Medical University School and Hospital of Stomatology, Taiyuan, 030001 China; 2 Shanxi Province Key Laboratory of Oral Diseases Prevention and New Materials, Taiyuan, 030001 China

**Keywords:** Extracellular vesicles, Drug delivery, Targeting, Nanoengineering EVs, Biodistribution

## Abstract

Precision medicine has put forward the proposition of "precision targeting" for modern drug delivery systems. Inspired by techniques from biology, pharmaceutical sciences, and nanoengineering, numerous targeted drug delivery systems have been developed in recent decades. But the large-scale applications of these systems are limited due to unsatisfactory targeting efficiency, cytotoxicity, easy removability, and instability. As such, the natural endogenous cargo delivery vehicle—extracellular vesicles (EVs)—have sparked significant interest for its unique inherent targeting properties, biocompatibility, transmembrane ability, and circulatory stability. The membranes of EVs are enriched for receptors or ligands that interact with target cells, which endows them with inherent targeting mission. However, most of the natural therapeutic EVs face the fate of being cleared by macrophages, resulting in off-target. Therefore, the specificity of natural EVs delivery systems urgently needs to be further improved. In this review, we comprehensively summarize the inherent homing mechanisms of EVs and the effects of the donor cell source and administration route on targeting specificity. We then go over nanoengineering techniques that modify EVs for improving specific targeting, such as source cell alteration and modification of EVs surface. We also highlight the auxiliary strategies to enhance specificity by changing the external environment, such as magnetic and photothermal. Furthermore, contemporary issues such as the lack of a gold standard for assessing targeting efficiency are discussed. This review will provide new insights into the development of precision medicine delivery systems.

**Graphical Abstract:**

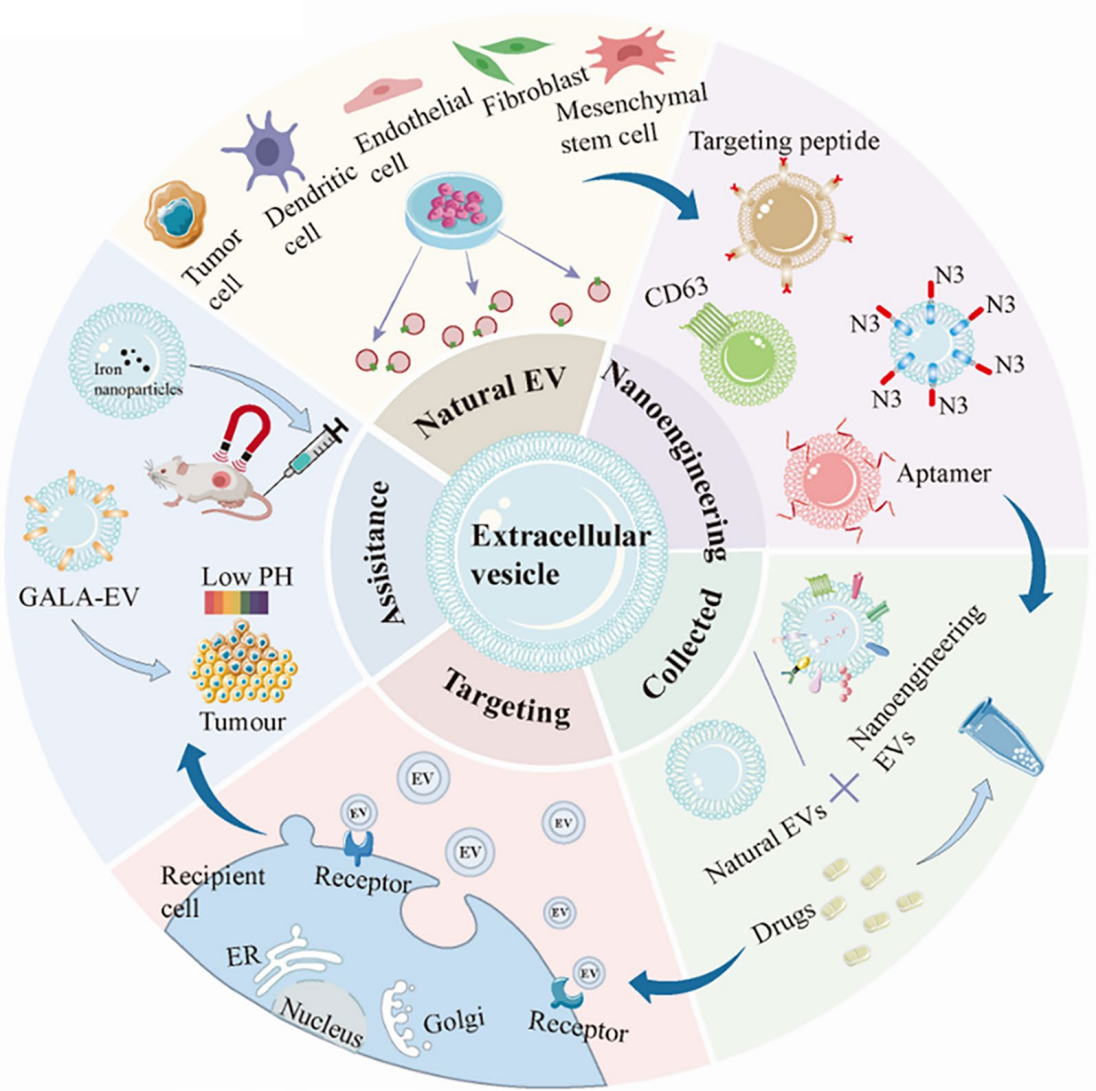

## Highlights

1. Tracing the full range of factors affecting EVs targeting mission in terms of EV components, cell sources, and delivery methods.
2. Strategies for EVs surface display and post-isolation methods facilitate the targeted delivery efficiency of the loading therapeutic cargo.
3. EVs complemented by non-invasive strategies such as magnetic fields, ultrasound, and photothermal further enhance targeting efficacy.

## Introduction

Precision medicine poses new challenges for modern drug delivery systems, the most important of which is targeted precision delivery [1]. Although researchers have spent decades developing targeted drug delivery systems, they still face challenges such as their side effects and failure to deliver precisely on target, being blocked by tissue barriers, being eliminated by the body as a foreigner [2–4]. Extracellular vesicles (EVs), 50–1000 nm nanovesicles that play roles in near and distant-range intercellular communication, are natural cargo carriers in our bodies [5–7]. Due to limited isolation techniques, EVs are currently divided into microvesicles, exosomes, and other EV subgroups [8], which differ in size, mode of production, and contents, and we focus on the EVs targeting drug delivery systems in this review. Researchers have used it as a novel drug delivery tool because of several properties: (1) excellent biocompatibility and low immunogenicity [9, 10]; (2) the internal vesicle structure provides drug-carrying space and protects the internal material from degradation in circulating tissue fluid [11]; (3) the external lipid bilayer allows it to cross various tissue barriers (99% of molecules are blocked) [12–15]; and (4) the proteins, lipids, and glycans on the membrane surface give it homing mission and provide natural sites for artificial modifications [16, 17]. As such, if the targeting specificity can be improved further, EVs are expected to be a milestone step for covering the current shortage in targeted precision drug delivery. It is imperative to explore its homing mission and develop solutions to improve its targeting ability.

The main equipment for EVs to accomplish targeted missions is structures enriched on the surface of EVs, such as receptor-ligand proteins. The surface enrichment of EVs with numerous receptors or ligands that have a natural affinity for receptors heavily expressed on the surface of target cells is the method by which they are targeted [18]. Therapeutic drugs or nucleic acid molecules are distributed to tissues or organs employing EVs as transporters when they are released into the extracellular matrix [19, 20]. Numerous studies have demonstrated that released EVs tend to convey specific molecules to the parental cells, which aids EV homing [21]. However, because not all EVs are directed at source cells, researchers need tailor their parental cell selection. Furthermore, different delivery methods can impact EVs distribution, and understanding these characteristics can help to reduce adverse effects and boost EVs bioavailability.

Unfortunately, the surface shape, cellular origin, and mode of administration targeting missions provided to EVs are insufficient to contend with the complicated in vivo microenvironment. Structural fragments such as proteins on the surface of EVs are expected to customize switches for their targeting capabilities. Nano-engineered EVs are those created by changing EVs’ parent cells or directly on EVs [22, 23]. For example, presenting nested peptides or antibody fragments on the surface of EVs to obtain targeted EVs [24, 25], as well as direct modifications to the EV surface, including click chemistry [26] and developing a membrane anchoring platform called "cloaking" to facilitate uptake by target cells [27]. Furthermore, by adding a second switch to EVs, it can be employed to increase EV accumulation in target organs using external auxiliary strategies, including magnetic and photothermal effects [28, 29]. These efforts usher in a new era of EVs-targeted drug delivery systems.

In this review, we provide a comprehensive description of the natural homing mechanism of EVs, starting from the enhanced permeability and retention (EPR) effects of EVs in targeting tumor cells and the specific targeting molecules on the surface of EVs. Furthermore, we discussed the effects of various cell sources and administration routes on EVs reaching the target cells. We highlighted and analyzed numerous methodologies for genetic alteration and direct modification of EVs in the realm of nanoengineered EVs targeting technology. The use of magnetic fields, ultrasound, photothermal and PH-sensing peptides as targeting aids to facilitate the effective accumulation of EVs at the site of injury. Finally, we describe some difficulties associated with EV-targeted drug delivery design systems.

## Mission: what determine where the EVs to go?

### The components of EVs determine their biodistribution

Tetraspanins are often viewed as surface markers of EVs and are involved in processes such as cell activation and adhesion [30, 31]. For example, tetraspanin-8 (Tspan8) is localized on the EV surface and forms a complex with integrin α4 or CD49d to target CD54-mediated endothelial and pancreatic cells [16, 32]. Another transmembrane protein focused on in the targeting process is integrins. Various combinations of α and β subunits result in different expression forms of integrins, which affect the specific uptake of EVs by target cells [33]. Their binding to target cells is an essential factor in EVs leading to tumor spread in most cases. Ligands highly expressed on the surface of receptor cells, such as a large amount of transferrin (Tf) on the surface of cancer cells, can bind to transferrin receptors (TfR) naturally present on the surface of EVs [34]. Furthermore, milk-derived EVs also express specific proteins on their surface that contribute to targeting uptake. This fleshes out the role of proteins in the specific uptake of EVs of multiple cellular origins. Milk-derived EVs naturally express soluble mucin 1 (MUC1) and lymphocyte function associated antigen-1 (LFA-1) as ligands for DC-SIGN (dendritic cell-specific ICAM-grabbing non-integrin) and ICAM-1 (Intercellular-adhesion molecule 1), respectively [35]. Based on this, Näslund et al. found that milk-derived EVs target monocyte-derived dendritic cells partly through the interaction between DC-SIGN and MUC1 [17]. Glycan modification of milk-derived EVs affects EV capture by human intestinal cells [36].

**Fig. 1:**
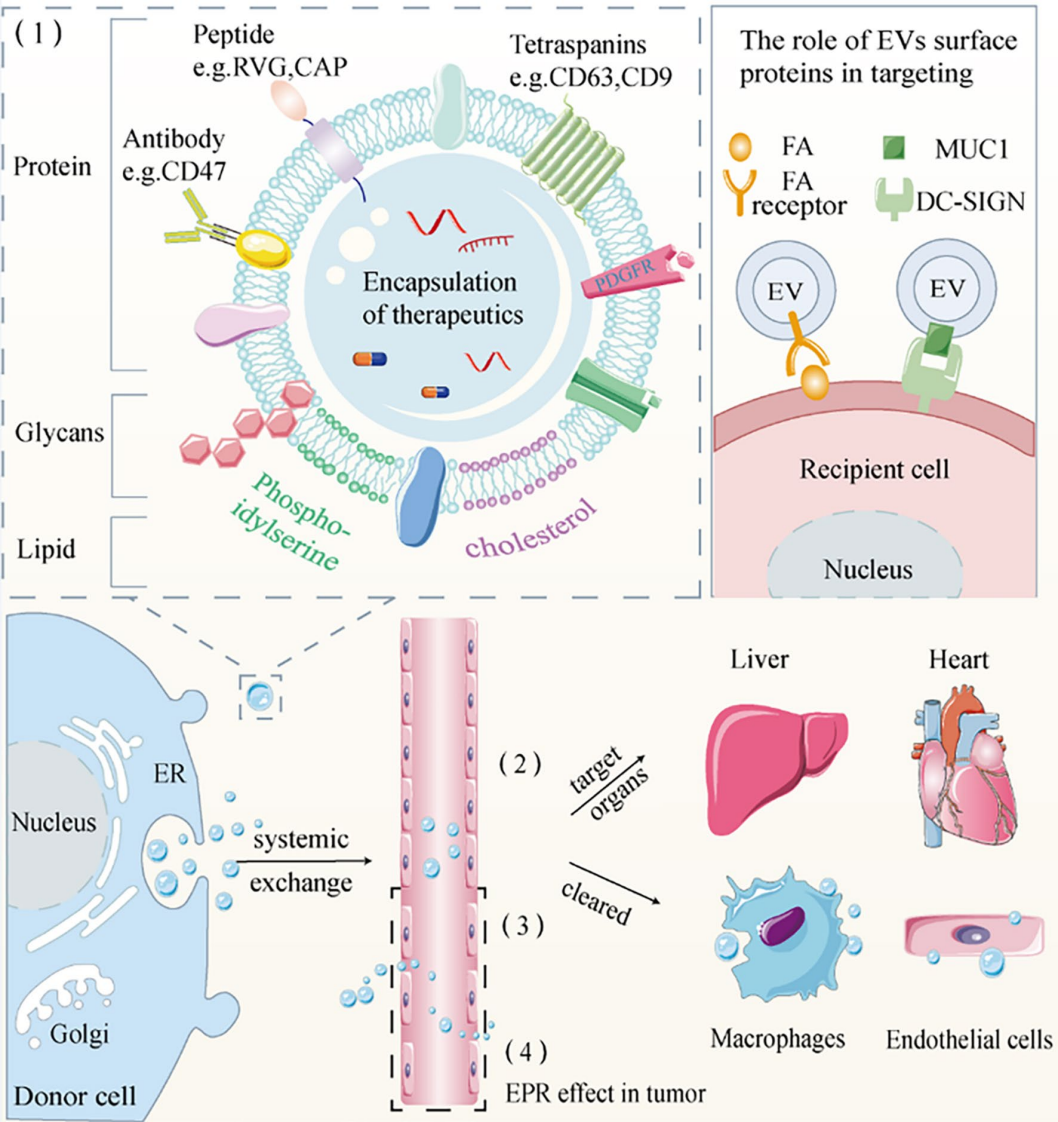
The inherent homing mechanism of EVs. (1) Targeting molecules on the surface of EVs; (2) EVs have natural organ tropism; (3) EVs are phagocytosed by macrophages and endothelial cells when they enter the circulatory system; (4) EVs have an EPR effect in tumor cells due to their nanoscale diameter

The phospholipid bilayer of EVs, composed of phosphatidylserine (PS), cholesterol and sphingolipids, is also responsible for the process of EVs targeting to specific cells. For example, PS can target immune cells by binding to the phosphatidylserine receptor (PSR) that causes immunosuppression, but more often, PS is recognized by macrophages and leads to EV clearance [37, 38]. Flannagan et al. demonstrated that the T-cell immunoglobulin mucin 4 (TIM4) can also specifically bind PS to be engulfed by macrophages [39]. Matsumoto et al. found a 66% reduction in phagocytosis of EVs by macrophages after cloaking the PS on the EV surface by annexin V (AnV) [40]. The researchers further explored and concluded that this recognition effect is mainly dependent on the negative charge on the PS surface [40]. Finally, glycans on the surface of EVs also bind to the chemokine (C–C motif) receptor 8 (CCR8) and the soluble ligand chemokine (C–C motif) ligand 18 (CCL18), targeting cancer cells [41]. Thus, the identification of specific molecules on the surface of EVs is the most critical part of the targeted therapy chain (Fig. 1).

Due to the removal of macrophages and the reticuloendothelial system (RES) [42], EVs are often off-target and fail to reach their destination. Therefore, reducing this off-target effect is essential to improve the bioavailability of targeted tissues. In addition to the series of molecules expressed above that promote EVs-specific uptake, molecules that reduce off-target effects. CD47 is an integrin protein widely found on the surface of EVs that reduces phagocytosis by macrophages and improves circulatory stability. Upon the release of EVs into the circulatory system, CD47 readily binds to signal regulatory protein-α (SIRPα) on the macrophage surface, releasing a "don’t eat me" signal [43]. Belhadj et al. proposed a new targeting strategy named "eat me/don’t eat me" [44]. Reduced EVs binding to macrophages means that more EVs can reach the target cells/tissues.

When focusing on tumor cells in receptor cells, we found that natural EVs have been extensively studied for targeting tumors, which is largely attributed to the EPR effect [45, 46]. Specifically, the small diameter of EVs allows them to be retained on the surface of tumor endothelial cells in close proximity to loosely arranged blood vessels (up to 500 nm) [47]. Follow-up experiments have demonstrated that the passive accumulation of EVs within solid tumors is not limited to this simple mechanism, but also depends on the role of immune cells within the tumor [48]. Zhang et al. designed a series of targeted drug delivery systems based on principles including the EPR effect, which included tumor stem cell-derived EVs, ultimately achieving preferential accumulation of drugs within the tumor. The EPR effect can be combined with active targeting strategies to achieve further targeting complementary effects [49]. However, the EPR effect is limited by patient-to-patient heterogeneity and is more appropriate for xenograft models [48, 50].

### Cell sources point the way for EVs to go

**Fig. 2:**
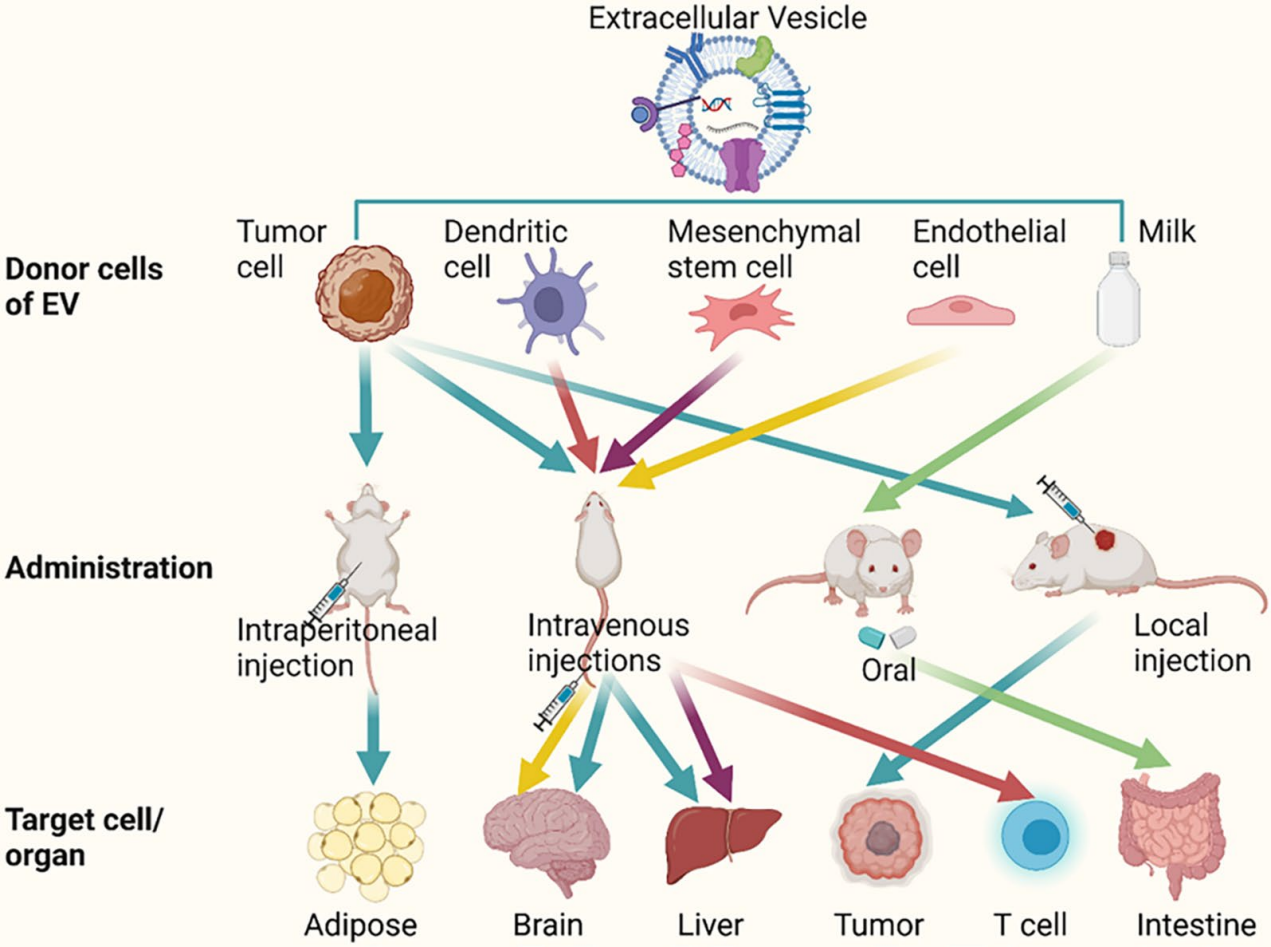
The way EVs target specific cells or tissues. Different donor cells and injection methods affect EVs targeting. Secreted EVs often have the characteristics of parent cells, so most EVs choose to home to cells or tissues that are histologically similar. Most EVs reach the liver via intravenous injection, the adipose tissue via intraperitoneal injection, specific sites such as tumors via local injection, and the intestine via oral administration. Select EVs donor cells and injection methods according to different needs. (Created with BioRender.com.)

The above targeting effect is mainly determined by the molecules on the surface of EVs, and EVs inherit some proteins from parental cells when they are secreted [51], so the cell source is an important factor influencing targeting (Fig. 2). EVs prefer to be found in cells histogenetically close to them: tumor cells, schwann cells, and microglia aim to homologous tumor cells, peripheral nerve, and central nervous system, respectively [52–54]. Moreover, the lower pH of the injured tissue provides a favorable environment for EVs targeting [55]. The parental cells that produce EVs can thus be chosen based on the condition to be treated.

#### Tumor-derived EVs

Due to the organ tropism of extracellular vesicles, most tumor-derived EVs will home to the tumor cells themselves. Based on this feature, EVs can be used as a powerful tool for targeting cancer therapy. Cancer cell membrane antigens E-cadherin is expressed on the surface of urine-derived EVs from prostate cancer patients, which allows EVs to preferentially target homologous tumors [56, 57]. Li et al. isolated tumor cell-derived EVs loading with the anticancer drug doxorubicin (Dox), and injected them into mice intravenously. They found that tumor cell-derived EVs localized to tumors more efficiently, while 64.86% of HeLa cell-produced EVs accumulated in the liver. Moreover, the parental cell tropism of cancer cell-derived EVs was twice as high as that of HeLa cells [52]. Human colorectal cancer cell-derived EVs loaded with anticancer drugs demonstrate concentration-dependent intra-tumor cell accumulation [58].

However, tumor-derived EVs also target other sites, such as immune cells, and endothelial cells. Molecules on tumor-derived EVs that achieve natural targeting include tumor antigens expressed on their surface, which are presented to immune cells as specific stimuli and mediate anti-tumor effects. Chen et al. demonstrated that B lymphoma cell-derived EVs containing heat shock proteins (HSP60 and HSP90) and major histocompatibility complex (MHC) molecules enabled them to act as antigen presentation vehicles, targeting T cells and exhibiting more potent immunostimulatory activity [59]. The presence of integrins inclines melanoma-derived EVs to be distributed in the brain and liver. Because of the metastatic nature of tumors, visual tracking of tumor-derived EVs reveals that they eventually preferentially tend to accumulate in organs such as the liver [60].

#### Immune cell-derived EVs

Dendritic cell (DC) -derived EVs express MHC on their surface and trigger immune effects [61]. It was shown that dendritic cell-derived EVs can home in on themselves, allowing MHC molecules to be expressed on the surface of dendritic cells and presented to T cells [62, 63]. Segura et al. further demonstrated that mature DC-derived vesicles can express MHC antigen complexes more efficiently compared with immature DCs [64]. Kowal et al. specifically reviewed the journey of DC-derived EVs to reach target cells [18].

LFA-1 is a part of integrins and is involved in cell adhesion and T-cell activation [65]. LFA-1 is present on the surface of EVs produced by macrophages. ICAM-1 is a ligand for LFA-1 acting in the immune process [66]. Yuan et al. exploited this specific binding to achieve targeting of macrophage-derived EVs to cerebrovascular endothelial cells, a process in which C-type lectin receptors also mediated the uptake of EV. Compared to controls, EVs accumulation in the brain was increased 3.6-fold [67]. In addition, T cell-derived EVs could target endothelial cells through the interaction between platelet-reactive protein-1 and cluster of differentiation 47 (CD47) [68]. EVs produced by stimulation of B cells using CD40 and interleukin-4 (IL-4) can be delivered to macrophages or hepatocytes as carriers of miRNA [69].

#### Mesenchymal stem cells-derived EVs

Mesenchymal stem cells (MSCs) have become an effective treatment for various diseases. After systemic injection, because of the large size of bone marrow mesenchymal stem cells (BMSCs), they quickly became trapped in the pulmonary vascular bed and typically less than 1% of BMSCs could be homed to the target site [70]. MSC-derived EVs offer new insights into alternative MSC-targeted therapies for a variety of diseases: such as type 2 diabetes due to insulin resistance [71], bronchopulmonary dysplasia [72], and neurodegenerative diseases [73]. MSC-derived EVs translocate to lymph nodes via surface glycans bound to lectins [74]. It has also been shown that MSC- derived EVs effectively accumulate in the liver (71% ± 4.0) with less accumulation found in the intestine (3% ± 1.5) [75]. It has also been demonstrated that MSC-derived EVs can inhibit STAT3 signaling and reduce pulmonary hypertension in a mouse model of hypoxic pulmonary hypertension, whereas fibroblast-derived EVs have no such effect [76]. Given the tumor tropism of MSC-derived EVs, researchers often encapsulate nucleic acids and drugs into vesicles to target and inhibit tumor growth. Naseri et al. used MSC-derived EVs loaded with LNA-anti-miR-142-3p to penetrate tumors to induce apoptosis [77]. Lou et al. used MSC-derived EVs-mediated miR-199a targeting to improve drug resistance in hepatocellular carcinoma [78].

#### Other sources-derived EVs

In addition to the abovementioned common EVs-producing cells, many other cells could be suitable sources for EVs-targeted therapy, including endothelial cells, blood, and milk. The lack of bone targeting strategies has been a bottleneck in the clinical management of degenerative bone lesions [79]. Endothelial cell-secreted EVs have a higher bone targeting capacity compared with other sources of EVs. Song et al. evaluated the bone-targeting ability of endothelial cells, bone marrow MSCs and osteoblast-derived EVs by observing the distribution of intravenously injected Dil-labeled EVs and found that only endothelium-derived EVs had fluorescent signals in bone [80]. The barrier permeability of EVs brings light to brain drug delivery, which is difficult to reach the target location due to the obstruction of the blood–brain barrier. Moreover, the abundant expression of transferrin receptors on the surface of blood-derived EVs can further facilitate the natural homing of EVs to the brain by binding to transferrin. Blood-derived EVs allow for a broader distribution of the drug in the brain, 15 times more than that of freely administered drugs [81–83]. Animal-derived EVs are approximately subject to safety concerns, so the use of milk as an EV carrier for targeting has started to become a focus of interest [84]. The surface glycoproteins of lactogenic EVs are important in specific recognition with target cells and EVs uptake [85]. Milk-derived EVs encapsulated with paclitaxel are stable, have low toxicity and exhibit significant targeting and tumor suppression [86].

### Administration route determines EVs targeting fate

Different routes of administration lead to different rates of drug arrival at the target organ and eventual accumulation in different organs. For example, when nitroglycerin is used to treat angina pectoris, sublingual administration is often chosen to reach the treatment site more rapidly. The EVs serve as vehicles for drug delivery, and the way they are injected is also critical for targeted therapy. In this section, we will focus on the effect of the mode of administration on the destination of the EVs (Fig. 2).

#### Intravenous injection

The most common mode of administration is intravenous, considering that intravenous injections allow direct access to the body circulation. Wiklander et al. evaluated the effect of the injection route on EVs distribution and found that intravenous injection accumulated more in the liver compared to other methods (60% ± 3.9) [75]. The results of Brossa et al. showed that intravenous injection of EVs derived from human liver stem cells was the optimal route of administration and significantly inhibited the growth of subcutaneous tumors [87]. In addition to this, there are many examples of tail vein targeted therapies that can target the liver, lung, spleen, kidney and heart [88–91]. However, the use of such injections often presents significant challenges, including drug accumulation in non-targeted tissues and problems with short half-lives (from minutes to hours) and rapid clearance rates.

#### Intraperitoneal injection

Intraperitoneal injection is also an effective method for EVs to deliver therapeutic molecules to target cells. Heidari et al. injected mesenchymal stem cell-derived EVs into the intraperitoneal cavity for treating acute colitis caused by dextran sulfate sodium [92]. Nojehdehi et al. demonstrated that intraperitoneal injection of adipose MSC-derived EVs stabilized blood glucose and effectively modulated immune responses compared with controls [93]. Zhou et al. compared intravenous and intraperitoneal administration of EVs and found that intravenous administration made EVs more likely to accumulate in organs that clear drugs, such as the liver, compared with intraperitoneal injection of EVs that exhibited a broad distribution within adipose tissue [94].

#### Oral administration

With the development of milk as one of the promising sources of EVs, oral EVs have become a more convenient and safer way to administer drugs without the need for a medical professional. Oral administration of milk-derived EVs can be used to observe improvements in joint inflammation. The data conclude that EVs in the treatment group do affect T-cell immune gut microbiota directly or indirectly through inflammation and are important for autoimmune arthritis in IL-1Ra^−/−^ mice and collagen-induced arthritic mice [95]. Nazmek et al. found that EVs modulating the gut microbiota with anti-OVA Ab FLC acquired antigen specificity and used EVs delivery of inhibitory miR-150 to target antigen-presenting cells to suppress delayed hypersensitivity reactions [96]. One researcher compared the distribution of EVs in vivo after intravenous and oral administration. The results showed that intravenous administration tended to be distributed in the liver and spleen, whereas oral administration tended to accumulate in the small intestine [97].

#### Local administration

In addition to the abovementioned injection methods, local delivery, particularly intratumoral delivery in cancer treatment, has received a lot of attention. This is because it allows the drug to concentrate on a specific site. Taking advantage of this feature, local injection of the drug facilitates its accumulation in the injured tissue. In the prostate cancer mouse model, intratumor-injected EVs were more bound to tumor tissue, and TNF-related apoptosis-inducing ligand (TRAIL^+^) EVs in cholesterol liposomes resulted in a significant reduction in tumor growth in mice (58%), which depended on the intratumoral EVs administration [98, 99]. Wang et al. observed in vivo the distribution of Dil-labeled extracellular vesicles by intratumor injection and tail vein injection, and showed that intratumor injection could more effectively increase local EVs concentration and inhibit tumor growth [100]. Despite the high delivery efficiency, this method is extremely invasive and often not easily accepted by patients. Moreover, there is a possibility of sudden drug release by local injection.

In summary, EVs are taken up by various routes, and specific routes of injection may result in different amounts of drug accumulating at different sites. Intravenous injection allows EVs to rapidly enter the body circulation and the procedure is simple, allowing the drug to reach and maintain therapeutic concentrations in a short period, but it is easy to off-target and is suitable for systemic diseases; intraperitoneal injection is suitable for metabolic diseases, and oral administration tends to target EVs to digestive system such as the small intestine, while local injection can solve the problem of low targeting efficiency, with rapid drug onset and few side effects, playing an irreplaceable role in tumor treatment. However, it sometimes leads to abnormal accumulation of EVs at a specific site. Therefore, the choice of drug delivery method should be based on the purpose of treatment and the site of targeting. Table 2 summarized the different sources of EVs targeted to various organs by suitable injection routes.

## Upgraded: what strategies contribute to the targeting ability?

The specific benefits of unmodified EVs are targeted. Unfortunately, this weak specificity is not always sufficient to achieve the targeted emission of the therapeutic payload. Scholars have turned their attention to nanoengineering EVs for improved targeting. Several approaches led to significant advances in targeted EVs delivery, such as genetic engineering, click chemistry, aptamers, and several complementary targeting strategies.

### Surface display

The natural targeting ability of EVs is based on the binding of EVs membrane proteins to receptors on the surface of the cell membrane, but this approach is limited by the need to find highly expressed receptors or ligands on the surface of the EV. Researchers have therefore devised a series of active targeting approaches to address this problem. Surface display means genetic modification of parental cells, and the classical approach is to transfect specific peptide fragments by fusing them to sequences encoding EVs transmembrane peptides, thereby allowing the protein of interest to attach to the vesicle surface. Transmembrane proteins on the surface of EVs, such as integrins, lactadherin (LA) and lysosome-associated membrane protein-2b (Lamp-2b), tetraspanins (CD9, CD63, and CD81), which can be combined with specific targeting ligands for enhancing their targeting ability. Lamp-2b, often found on dendritic cell-derived EVs, is a commonly used membrane protein that contributes to EVs homing [101]. Alvarez-Erviti et al. developed nanoengineering EVs by transfecting plasmids encoding the Lamp-2b constructs into the dendritic cells 4 days before EV purification, and then rabies viral glycoprotein (RVG) peptide was cloned into Lamp2b [24]. This strategy resulted in the generation of EVs exhibiting a higher tropism for the central nervous system. Liang et al. transfected dendritic cells with CAP-GFP-lamp2b plasmid to generate EVs targeting cartilage arthritis. They combined the superior tissue penetration capability of the EVs and the cell-specific targeting of a chondrocyte-affinity peptide (CAP) [25]. Cell-specific binding peptides can be fused to the extracellular domain of Lamp-2b at the N-terminus and be degraded by endosomal proteases during EVs biogenesis and secretion. To suppress peptide loss, a glycosylation motif (GNSTM) can be added to the N-terminus of peptide–Lamp-2b fusions [102].

In addition to Lamp-2b, other membrane proteins on the surface of EVs can serve as anchor points for targeting motifs, for example, cluster of differentiation 9 (CD9) can bind to human antigen R (HuR), an RNA binding protein [103]; the transmembrane protein platelet-derived growth factor receptor (PDGFR) can fuse with the GE11 peptides. Ohno et al. established a novel strategy to engineer the human embryonic kidney 293 (HEK293) cells to generate EVs expressing the GE11 peptide. EVs modified with GE11 peptide bind specifically to the epidermal growth factor receptor (EGFR), which is highly expressed on the surface of tumor cells and is a receptor target in the cancer drug delivery system [104]. The C1C2 structural domain can serve as a bridge between the PS and antigen linkage in EV membrane for targeting [105]. Thus, the design of a fusion of a targeting peptide such as Arg-Gly-Asp (RGD) peptide to the C1C2 structural domain would allow specific delivery of therapeutic EVs to the lesion area [106]. Table 1 demonstrates the use of various EV surface display protein-linked targeting peptides for treating of multiple diseases (Table 1).

**Table 1:** Surface-displayed proteins of EVs for targeted therapy

Protein on the surface of EV	Targeting peptide	Parent cell	Function	Effect	Measures of targeting efficiency	Refs.
Lamp-2b	RVG	Primary immature Dendritic cells	Alleviate Alzheimer’s disease	Central nervous system specific RVG binds specifically to acetylcholine receptor	Confocal laser imaging colocalization	[24]
	RGD	Leukemia cell line K562	Promoted therapeutic angiogenesis	Overexpression of αvβ3 integrin on the surface of blood vessels, which specifically binds to RGD peptide	Targeted metabolic labeling technique	[107]
	IMTP	BMSC	Enhanced vasculogenesis, and cardiac function	IMTP modified EVs can preferentially target ischemic myocardium	Near-Infrared Fluorescence Tracer	[108]
	CMP	CDCs	Modulating the cardiac remodeling	CMP modified EVs can preferentially target cardiomyocyte	Whole-organ fluorescence imaging	[109]
	RGE	Raw264.7	Inhibit glioma	Cross the BBB smoothly, accurately identify gliomas	Fluorescence imaging	[110]
	CAP	Dendritic cells	Alleviate OA progression	CAP peptide modified EVs can specifically target chondrocytes	Near-Infrared Fluorescence Tracer	[25]
	T7	HEK293T cells	Treatment of glioblastoma	T7 peptide has a higher efficiency of brain targeting than RVG peptide	Fluorescence imaging	[111]
	tLyP-1	HEK293T cell	Treatment for non-small cell lung cancer	tLyP-1 peptide can selectively target nerve NRP1 and NRP2	–	[112]
	E7	Dendritic cells	Alleviate OA progression	E7 peptide can target SF-MSC	Fluorescence imaging	[113]
	IL-3	HEK293T cell	Improve prognosis in CML patients	IL-3 can specifically bind to IL-3R overexpressed in CML	Fluorescence imaging	[114]
	Her2-binding affibody	HEK293T cell	Reverse the drug resistance in colorectal cancer	Her2-binding affibody binds to Her2 overexpressed on the surface of HCT-116^5FR^ cancer cells	Fluorescence imaging	[46]
PDGFR	GE11	HEK293T cell	Deliver let-7a to breast cancer tissues that highly express EGFR	The specific binding ability of GE11 peptide to cancer cells overexpressing EGFR is better than EGF	In vivo imaging system monitoring	[104]
CD63	myostatin propeptide	NIH3T3	Treatment for DMD	Propeptide can be anchored on the surface of EVs via CD63	Flow cytometric analysis	[115]
	OVA antigen	293-F cells	Improving DNA vaccine immunogenicity	OVA antigen fused to CD63 plasmid to produce EVs of antigen carriers that target CD8^+^ T cells to enhance immune response	–	[116]

RVG, Rabies viral glycoprotein; RGD, L-Asparticacid, L-arginylglycyl; IMTP, Ischemic myocardium-targeting peptide; BMSC, Bone Marrow Stromal Cells; CDCs, Cardiosphere-derived cells; CMP, Cardiomyocyte specific peptide; Raw264.7, Leukemia cells in mouse macrophage; BBB, Blood–brain barrier; CAP, Chondrocyte-affinity peptide; OA, Osteoarthritis; HEK293, Human embryonic kidney 293; SF-MSC, Synovial fluid-derived mesenchymal stem cells; NRP1, Neuropilin1; NRP2, Neuropilin2; IL3, Interleukin 3; CML, Chronic myeloid leukemia; HCT-116^5FR^, 5-FU-resistant derivative of the HCT-116 human colon cancer cell line; EGFR, Epidermal growth factor receptor; EGF, Epidermal growth factor; DMD, Duchenne muscular dystrophy; TRAIL, TNF-related apoptosis-inducing ligand

### Post-isolation methods

**Fig. 3:**
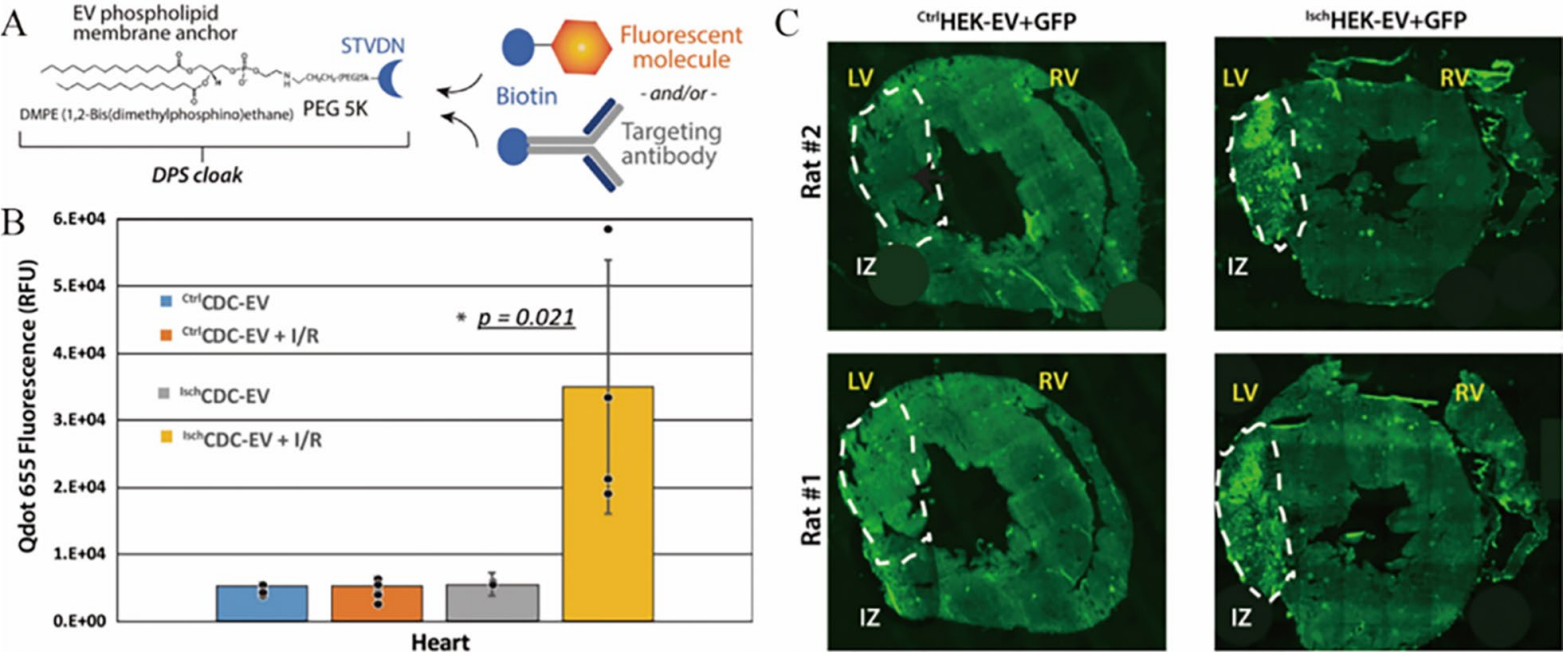
The clocking platform enhances the uptake of extracellular vesicles by the injured heart. **A** Schematic diagram of the construction of the clocking platform. **B** In the rat (I/R) model, ^Isch^CDC-EV exhibited significant cardiac targeting. **C** ^Isch^HEK-EV s exhibit enhanced targeting ability to damaged myocardium [27]. Copyright 2018, Journal of Nanobiotechnology

The above methods for modifying the parental cells of EVs suffer from low transfection efficiency, and researchers are currently working on further modifications of the secreted EVs to improve targeting efficiency. A nanoengineered EVs membrane targeting platform that also enables EVs to carry targeting peptides has been established, called cloaking (Fig. 3), in which biotinylated molecules can be linked to glycerol-phospholipid-PEG conjugates (DMPE-PEG) inserted into EVs phospholipid bilayers, thereby enhancing EVs uptake by damaged tissues. Antes et al. compared the cloaking with the surface display and found that there was no significant difference in targeting efficacy, and both had their own advantages [27], the specific limitations of which are discussed further below (see Section "Post-isolation methods").

Researchers have discovered an advanced method for improving the targeting function of EVs: chemically attaching some functional groups to the surface of EV membranes. Copper-catalyzed azide-alkyne cycloaddition (click chemistry) is an efficient and promising technique for introducing various functional azide groups into EVs for subsequent bioorthogonal reactions. Smith et al. applied this click chemistry approach and demonstrated that the method not only allows EVs to be more easily targeted to recipient cells by chemical bonding, but also does not alter the size of the EVs or affect the endocytosis of the target cells [26]. Jia et al. used click chemistry to bind EV membranes to RGE peptides and then produced engineered EVs called RGE-Exo-SPION/Cur with strong glioma targeting ability [110]. However, click chemistry is not applicable to somatic-derived EVs and is non-specific, which limits the recognition of EVs with target cells [117].

**Fig. 4:**
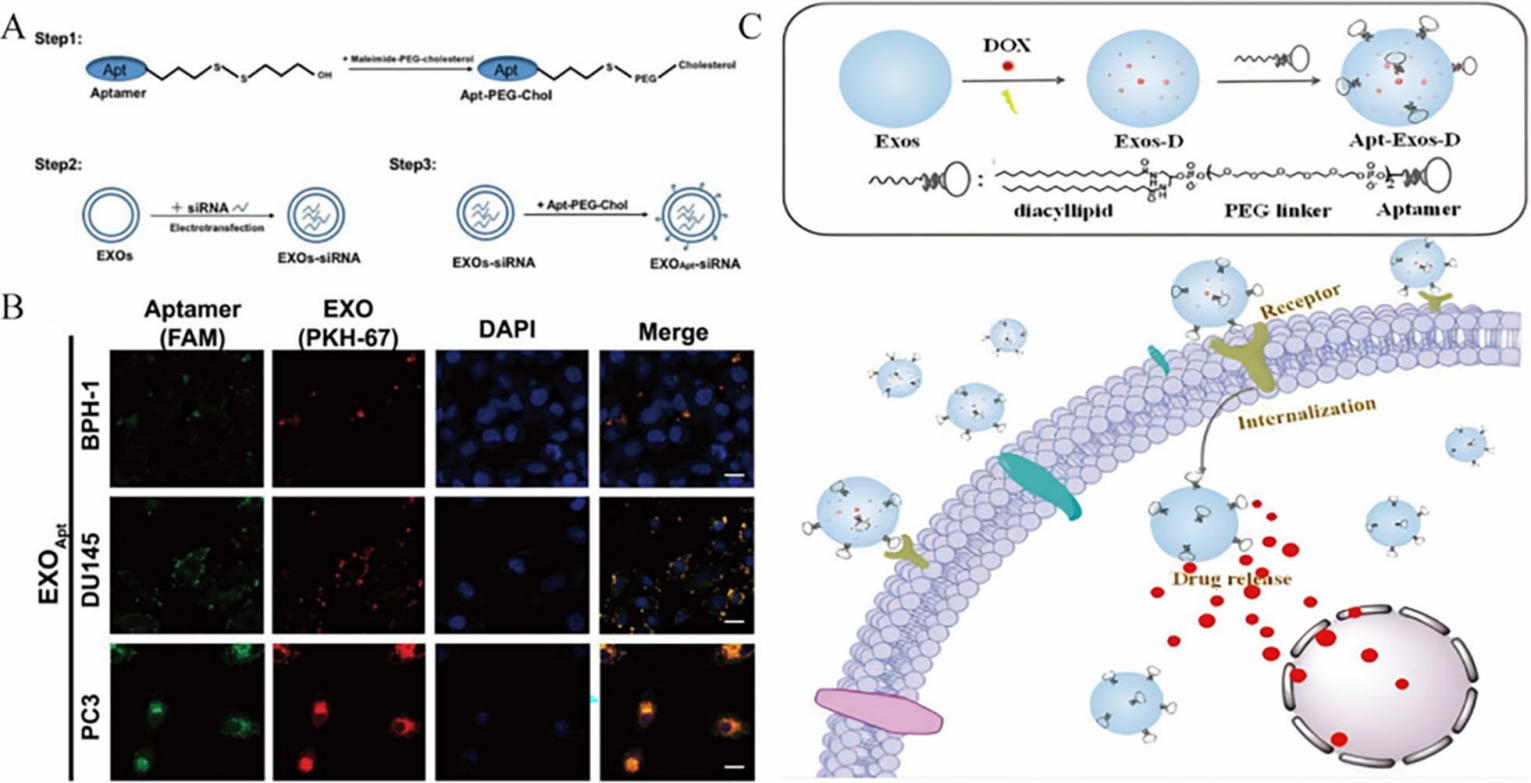
Aptamers as "chemical antibodies" for penetration and accumulation in tumors for targeted drug delivery. **A** Schematic diagram of the use of aptamer-PEG-cholesterol coupling to modify siRNA-loaded EVs (EXO_Apt-siRNA_). **B** Confocal images showing that EXO_Apt_ prefers to accumulate in prostate cancer cells [119]. Copyright 2021, Theranostics. **C** Use of Aptamer-Modified Dox-Encapsulated EVs (Apt-Exos-D) for effective drug delivery to target cancer [120]. Copyright 2019, Analytical Chemistry

Recently, a more effective, simpler and newer technology for actively targeted EV delivery has emerged: nanoengineering of aptamer-targeted EV delivery. Compared with the previously used antibodies and peptides, aptamers have some significant advantages, including higher stability, stronger specificity, less toxicity, and superb tissue penetration [118]. Considering the property that aptamers can bind to the corresponding ligands with high affinity, they have promising applications in the field of targeted therapeutics. E3 aptamers have been shown to specifically target prostate cancer cells in previous studies, so coupling E3 aptamers to the surface of EVs carrying siRNA reduced prostate cancer proliferation and metastasis both in vitro and in vivo [119] (Fig. 4). In addition, Zou and colleagues developed an Apt-Exos nanoplatform for targeted delivery of anticancer drugs in which the cancer-specific nucleic acid aptamer Sgc8 is modified on the exosome surface by hydrophobic interaction [120]. The ability of Apt-Exos to target delivery of DOX was subsequently verified by fluorescent labeling (Fig. 4). Xiang et al. compared the effects of aptamers and peptides on tumor penetration and found that epithelial cell adhesion molecule (EpCAM) aptamers can effectively penetrate tumors and improve circulatory stability [121]. Moreover, Macdonald and colleagues developed a dual-functional aptamer combining EpA and TfR, which successfully delivered the drug to the brain through the blood–brain barrier [122]. RNA aptamer and aptamer-binding protein (ABP) interactions can be used to actively enrich Cas9 ribonucleoproteins from different species into vesicles, and fusion of Com to the N-terminus and C-terminus of CD63 to further increase the activity of gene editing [123]. This contributes to reducing the off-target effects of gene editing. In terms of disease treatment, EVs and aptamers also play a critical role. For example, Tran et al. introduced aptamer-guided EVs diagnostics and therapeutics in detail based on the targeted EV drug treatment system [124]. The study by Luo et al. provided an Apt-bone marrow stromal cell (ST) -derived EVs complex, which is an aptamer-based bone-specific targeting method for EVs delivery [125]. RVG-EVs loaded with aptamers targeted α-synuclein aggregates and eventually save synaptic protein loss and neuronal death in the parkinson disease (PD) mouse model [126]. The most critical advantage of these aptamers, which are called "chemical antibodies," is that they don’t have to consider the differences between batches.

**Fig. 5:**
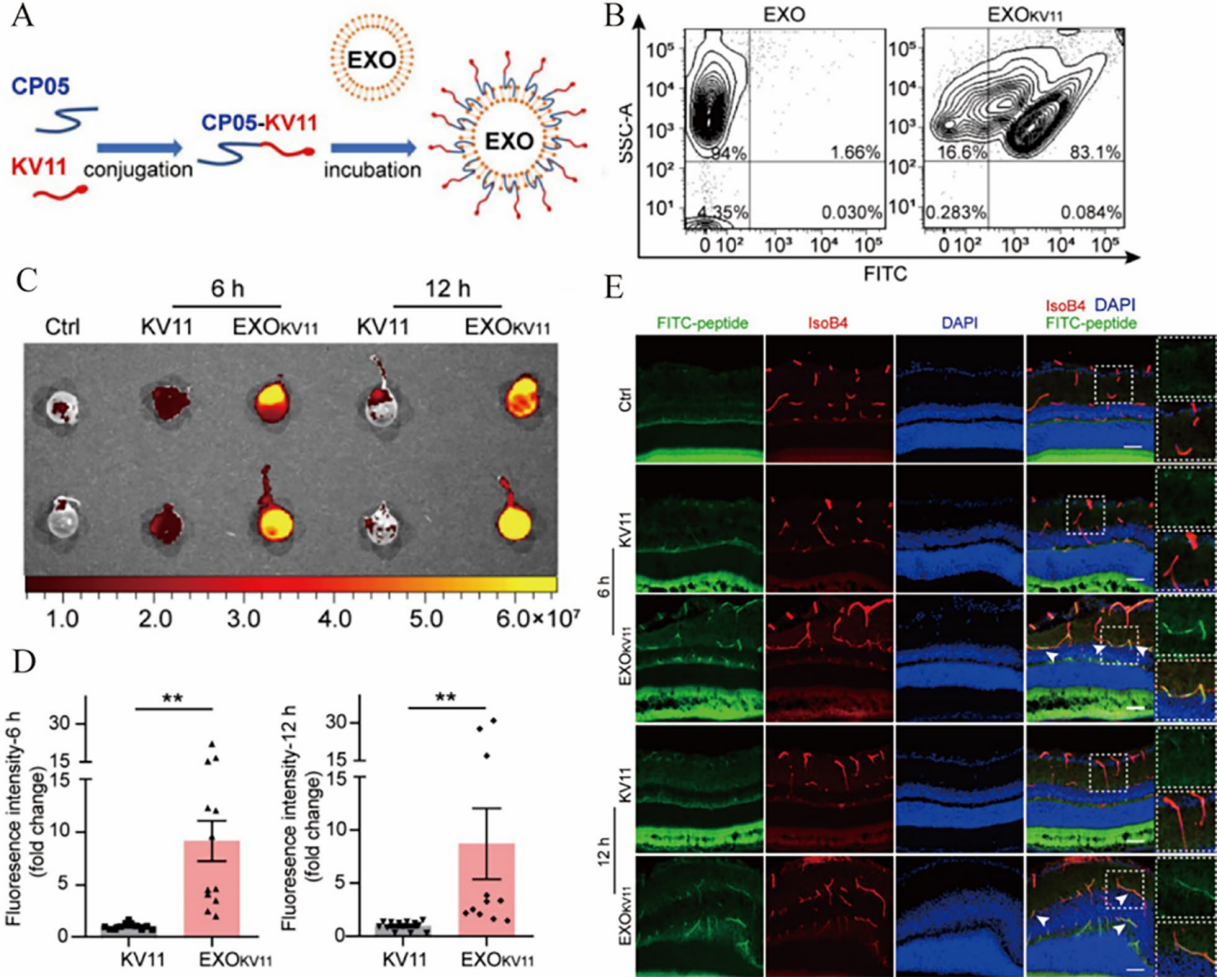
Through the specific binding of the CP05 peptide to CD63 on the surface of EVs, the anti-angiogenic peptide was bound to the surface of EVs to generate an EXOKV11 delivery system that targets retinal blood vessels. **A** Schematic illustration of the preparation of EXO_KV11_. **B** Flow cytometry results show that 83.1% of KV11 peptides were bound to EVs based on the effect of CP05. **C** Mice were injected with KV11 and EXO_KV11_, respectively, and treated with FITC labeling observed fluorescence. **D** Under the action of CP05, EXO_KV11_ entered the eye more efficiently compared with KV11 alone. **E** In the OIR mouse model, retinal cryosections shows that EXO_KV11_ targets retinal vessels more efficiently [128]. Copyright 2021, Theranostics

For precisely targeted drugs to treat disease, CP05 peptide was turned out. CP05 peptide can bind to CD63, a marker on the surface of EVs, with high affinity to the second extracellular loop (Fig. 5). Based on this, CP05 can capture EVs in the circulation and deliver a variety of cargos into specific tissues without any toxicity [127]. This method avoids the modification of therapeutic drugs into EVs, but directly fusion with CP05 peptide. CP05 was used as an anchor peptide to be fused with KV11 and incubated with EVs for 6 h to produce EXO_KV11_, which could efficiently deliver an anti-angiogenic peptide and suppresses neovascularization of the retina [128]. Similarly, using this method to anchor myostatin propeptide to the surface of EVs can improve muscle function and provide new possibilities for treating duchenne muscular dystrophy (DMD) [115].

Some features of the EV surface, such as negative charge, can also be used to produce targeted EVs. To make the EVs more aggregate in the liver tissue, Ryo Tamura et al. efficiently bound positively charged pullulan complexes to EVs with negatively charged surfaces via electrostatic interactions. The modification with cationized pullulan enhances the internalization of the modified EVs via asialoglycoprotein receptor (ASGPR) in hepatocytes or HepG2 cells. The disadvantage of targeting EVs based on electrostatic interactions is the cytotoxicity of pullulan [129]. After treating with positively charged polyethyleneimine (PEI), the intraocular lens surface can be targeted to fix with the negatively charged EVs by electrostatic interactions, and this method is effective in preventing posterior capsular opacification [130]. Nakase and colleagues reported a technique in which EVs were modified with GALA peptides and cationic lipids to effectively deliver macromolecular cargoes, such as dextran and saponin. Specifically, cationic lipids counteract some of the negative charges on the EV membrane surface and enhance their uptake by target cells, while GALA peptides also exhibit high affinity for target cell membranes under the low pH conditions of the tumor microenvironment [131].

The surface of EVs can be modified with the receptors of some targeting ligands to enhance their targeting capability. It is well known that folate (FA) receptor is overexpressed on the surface of the majority of cancer cells. Based on this, Zheng et al. constructed FA/exosome to deliver siRNA for effective cancer inhibition [132]. Yang et al. developed a pH-sensitive superparamagnetic nanoparticle cluster for the precise separation of blood EVs based on the specific recognition of Tf and TfR, and these pH changes do not cause damage to EVs [34]. The main drawback of this approach is the challenge and cost of synthesizing functional ligands rather than simple chemicals such as azide as in click chemistry [133]. With several strategies for nanoengineering EVs as described above, EVs are now achieving breakthroughs targeting multiple organs (Table 2).

**Table 2:** Multiple modified EVs encapsulate therapeutic cargo targeting various organs

Targeted organs	EV source	Modification	Therapeutic cargo	Injection method	Outcome	Refs.
Heart	CPCs	Transfection of miR-322 by electroporation after isolation of exosomes	miR-322	Tail intravenous injection	CPC-EV loaded with miR-322 can treat ischemic cardiovascular disease	[134]
	BMSC	Transfection of Lamp-2b with IMTP plasmid	Targeting peptide IMTP	Intravenous injections	IMTP-EVs are more easily taken up by hypoxia-injured cells, improving myocardial function	[108]
	CDCs	Transfection of Lamp-2b with CMP plasmid	Targeting peptide CMP	Intramyocardial injections and retroorbital intravenous injection	CMP-EVs improve selective targeting of ischemic heart tissue	[109]
	BMSCs(with hypoxic conditions)	Combine with IMT peptides through bio-orthogonal chemistry	miR-125b-5p	Intravenously injection	Improve the targeting efficiency of EV, reduce cardiomyocyte apoptosis and reduce off-target	[135]
	MSC	Platelet membranes to envelope EVs	–	Intravenous injections	Significantly increase the uptake of EVs by endothelial cells and cardiomyocytes, and reduce the phagocytosis of macrophages	[136]
Brain	MSC	Use click chemistry to couple functional ligands to the EV surface	Cur	Tail intravenous injection	cRGD-EV targets the ischemic brain injury area and inhibits inflammation and apoptosis in this area	[137]
	HEK 293 T cell line	Transfection of RVG peptide plasmid and mu (MOR) siRNA into donor cells	MOR siRNA	Intravenously injected	RVG EVs encapsulating MOR siRNA cross the BBB and target the central nervous system to treat drug addiction	[138]
	whole blood of SD rats	Coupling mAb GAP43 to the surface of Que-EV using the carbodiimide method	Quercetin	Tail intravenous injection	Que/mAb GAP43-EV enhances the accumulation of Que drugs at the site of cerebral ischemia injury and reduces the infarct size	[139]
	Neutrophil	–	Dox	Intracardiac injection; intravenously injected	NEs-EVs carries Dox across the BBB to target brain tumors	[140]
	Raw264.7	Combine RGE peptides with EVs loaded with SPION and Cur by click chemistry	SPIONs and Cur	Tail intravenous injection	RGE-EV-SPION/Cur can cross the blood–brain barrier, accurately identify and treat gliomas	[110]
Lung	BMM	Combine the mixture of EVs and DSPE-PEG-AA using ultrasound and PTX	PTX	Tail intravenous injection	AA-PEG-EV specifically delivers PTX to lung metastases	[141]
Bone	Dendritic cells	CAP-EV was synthesized by transfection of CAP-lamp2b plasmid	miR-140	Intraarticular injection	CAP-EVs remain in the joint cavity, deliver miR-140 to deep cartilage regions, and alleviate OA progression	[25]
	Endothelial cells	–	miR-155	Intraperitoneally injection	EC-EV is specifically taken up by BMMs, among which miR-155 inhibits osteoclast activity and reverses osteoporosis	[80]
	NIH-3T3 cells	Plasmid transfection enables high expression of CXCR4 on the surface of EVs	Antagomir-188	Tail intravenous injection	CXCR4-positive EVs could be recruited to the bone marrow	[79]
Skin	Human adipose tissue-derived mesenchymal stem cell	–	–	Intravenously or subcutaneously	Topical application	[142]
Liver	MSC	The EVs modified with cationized pullulan	–	Tail intravenous injection	The modified EVs accumulated in the liver tissue, resulting in a greater therapeutic effect on liver injury	[128]
Breast	imDCs	Transfect iRGD peptide binds to the exosomal surface membrane protein Lamp-2b	Dox	–	iRGD-EVs-Dox can be specifically delivered to the breast, playing a highly effective anti-tumor effect	[143]
Colon	Raw264.7	Produce HDEA@EVs including PH response HDEA, hyaluronic acid and Dox	Dox	Tail intravenous injection	HDEA@EVs specifically binds to the CD44 receptor on the tumor surface and delivers Dox to colon cancer tumor cells	[144]

CPCs, Cardiac progenitor cells; BMSC, Bone Marrow Stromal Cells; IMTP, Ischemic myocardium-targeting peptide; CDCs, Cardiosphere-derived cells; CMP, Cardiomyocyte specific peptide; MSC, Marrow Stromal Cells; RGD, L-Asparticacid,L-arginylglycyl-; Cur, Curcuminoid; RVG, Rabies viral glycoprotein; HEK293, Human embryonic kidney 293; MOR, Opioid receptor Mu; BBB, Blood–brain barrier; SD rat, Sprague–dawley rat; GAP43, Growth-associated protein-43; Que, Quercetin; Raw264.7, Leukemia cells in mouse macrophage; Dox, doxorubicin; NE, Neutrophil; SPIONs, Superparamagnetic iron oxide nanoparticles; RGE, Neuropilin-1-targeted peptide; BMM, Bone-marrow derived macrophages; PTX, Paclitaxel; AA-PEG, Aminoethylanisamide-polyethylene glycol; CAP, Chondrocyte-affinity peptide; OA, Osteoarthritis; EC, Endothelial cells; CXCR4, C-X-C motif chemokine receptor 4; imDC, immature dendritic cells; HDEA, Hyaluronic acid grafted with 3-(diethylamino)propylamine

### Limitations of various modification methods

**Fig. 6:**
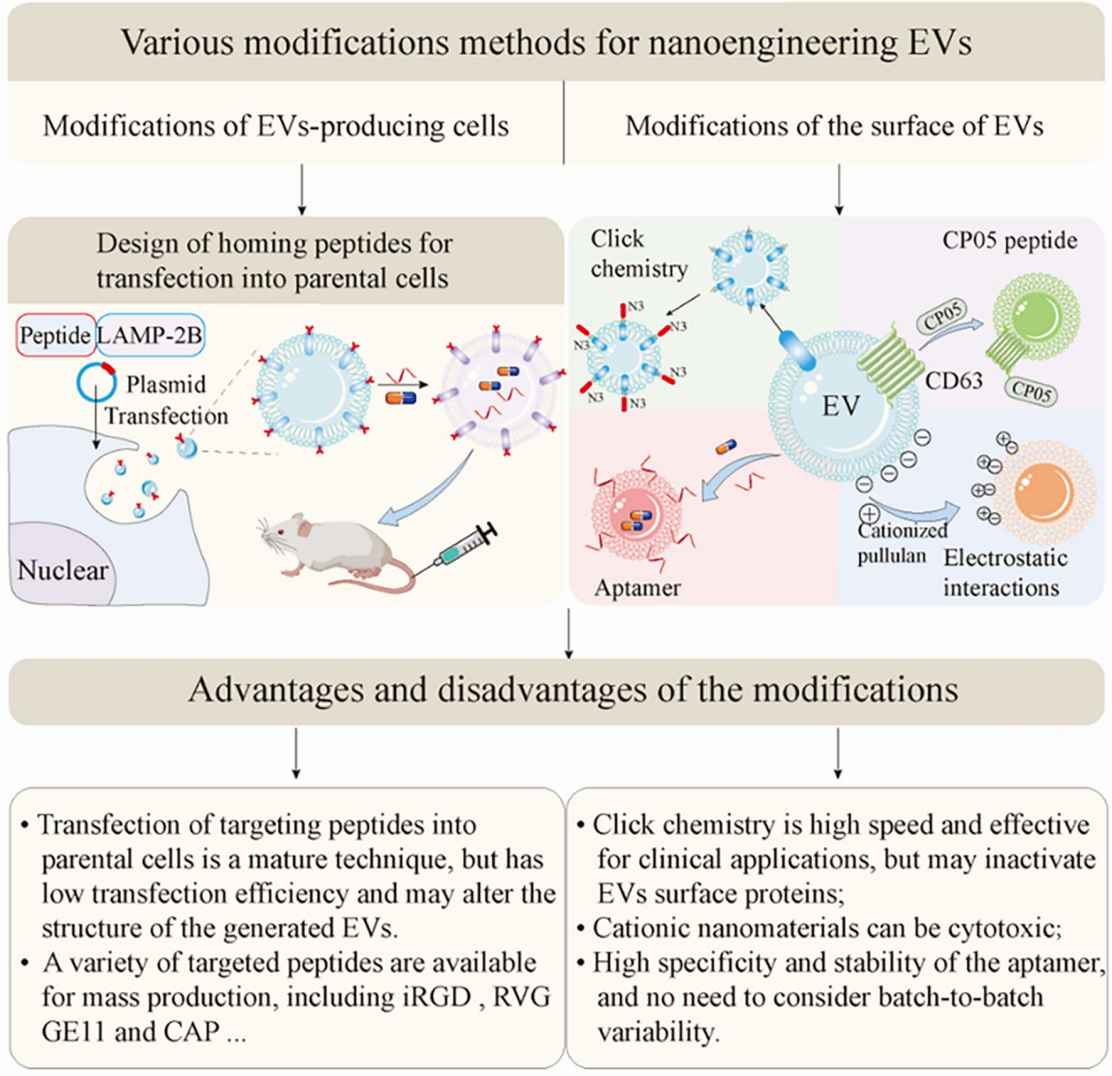
Design methods and characteristics of targeted EVs. EVs can be nanoengineered by modifying parental cells as well as by directly modifying isolated EVs. By fusing the peptides of interest into plasmids and transfecting them into donor cells, the secreted EVs express specific proteins and bind to recipient cells through receptor-ligand interactions (left). Once the EVs are isolated, they can be modified by click chemistry, CP05 peptides, aptamers, electrostatic interactions, and other strategies (right). Therapeutic cargoes can also be encapsulated into nanoengineered EVs. Finally, the advantages and disadvantages of various design approaches are presented so that researchers can choose the appropriate design

Although nanoengineered EVs have been extensively tested for targeted therapies, there are limitations to their various approaches. The typical approach to nanoengineered EVs is to transfect genes into the parental cells of the EVs so that the secreted EVs membrane expresses the target protein. The EVs subsequently bind specifically to receptors that are highly expressed at the site of the lesion. However, this gene modification method has some problems, such as safety issues, complex and expensive gene transfection, and unstable transfection, targeting peptides may affect the structure of the EVs surface [118]. One limitation of expressing antibodies on the EVs surface for targeting is the need to use known, validated single-stranded variable fragment sequences to fuse to a display domain such as C1C2 and express in the generating cells. L17E peptide was shown to increase the release of antibodies for targeting the cytosol [82]. The advantage of using EVs of specific parental cell origin is that the secreted EVs carry some of the properties of the parental cells, however, the modification of the secreted EVs can alter or even lose these endogenous properties [145]. The use of click chemistry allows a selective, rapid and effective method that is independent of the origin of the EV cells and is suitable for clinical applications but may inactivate EV surface proteins [146]. The overall negative charge of EVs is critical for uptake by target cells, and targeting approaches developed based on multiple charge interactions can reduce non-specific uptake by receptor cells. Current studies show that cationized pullulan can accelerate endocytosis, but the concern is the cytotoxicity of cationic nanomaterials and the risk of lysosomal degradation after loading is endocytosed into the receptor cell [147] (Fig. 6).

## Assistance: what external factors facilitate targeting effectiveness?

### Magnetic therapy

Surface modification of EVs has already yielded many remarkable results in improving targeting specificity, and surprisingly, researchers have found that targeting effects will be aided by synergistic external stimulation. Several researchers have considered the use of external magnetic fields to enhance the targeting of EVs. For example, magnetically functionalized blood EVs can be effectively targeted to treat cancer by preparing superparamagnetic iron oxide nanoparticles (SPIONs). The surface of EVs produced by reticulocytes within blood contains membrane proteins including TfR that can bind to superparamagnetic nanoparticles coupled to Tf. [28] Harvesting SMNC-EXO based on superparamagnetic magnetite colloidal nanocrystal clusters (SMCNCs) after loading Dox in the presence of magnetic fields (MFs) increases its internalization by tumors [148]. Kim et al. prepared MSC-generated magnetic nanovesicles, and encapsulated iron oxide nanoparticles (IONP) into the vesicles, which can target the site of cerebral ischemic injury with the assistance of a magnetic field [149]. Labeling astrocyte-derived EV with ultrasmall superparamagnetic iron oxide (USPIO) by intranasal perfusion can be distributed to the brain and produce fewer side effects. This provides a new idea for EV-targeted treatment of central system diseases [150].

### Photothermal therapy

**Fig. 7:**
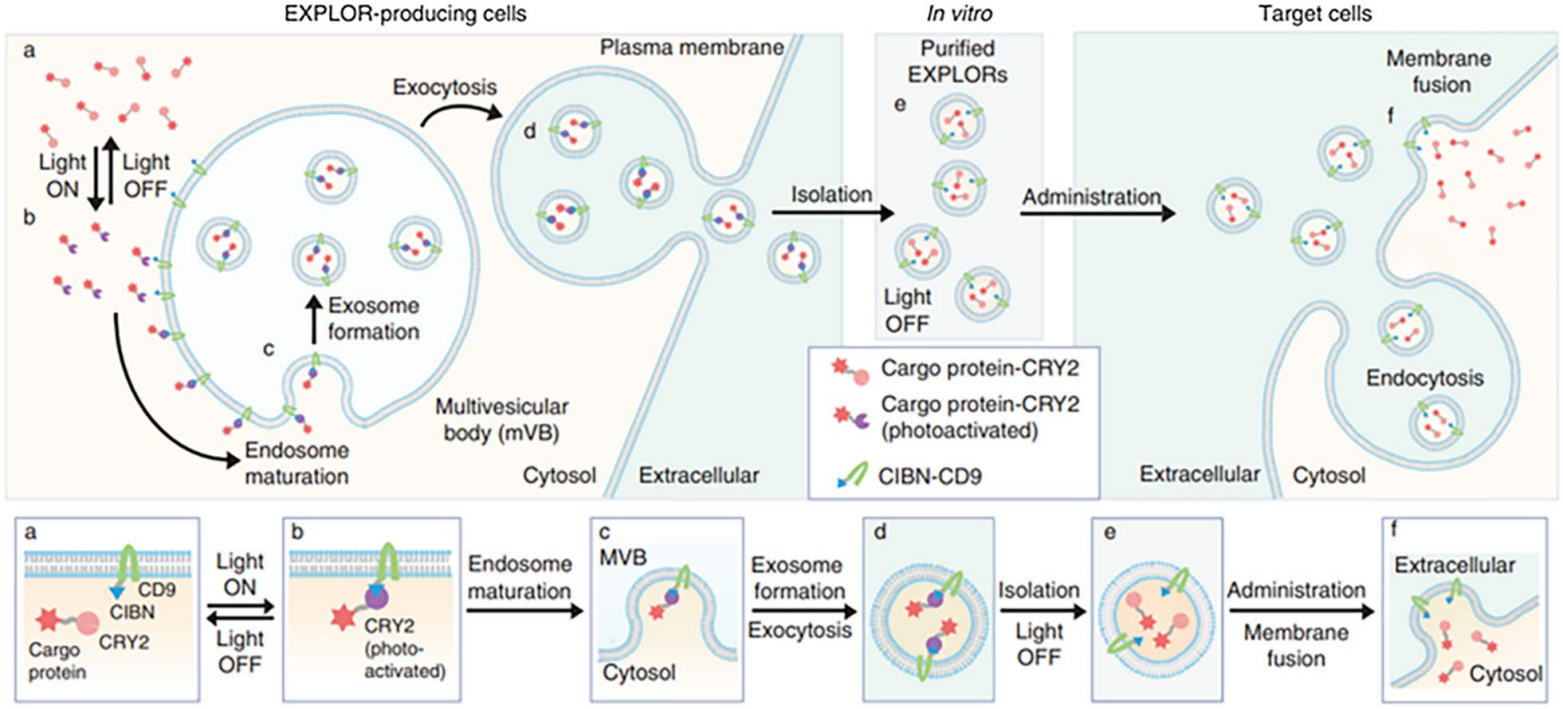
Schematic diagram of EXPLOR technology. In EXPLOR-producing donor cells, CRY2 protein was fused to a cargo protein, and CIBN was conjugated with a representative marker of EVs, CD9 protein. Blue light illumination induces the reversible PPI between CIBN and CRY2 fusion proteins. With continuous blue light irradiation, the cargo proteins are guided to the inner surface of the cell membrane or the surface of early endosomes. Mature multi-vesicular bodies (MVBs) then readily secrete cargo protein-carrying EVs (EXPLORs) from the cells by membrane fusion with the plasma membrane. After exocytosis, EXPLORs can be easily isolated and purified in vitro. Purified EXPLORs can be used for delivery of the cargo proteins into target cells via membrane fusion or endocytosis processes. Bottom grey boxes highlight the essential steps from EXPLORs biogenesis to target cell delivery [152]. Copyright 2016, Nature Communications

To achieve precise treatment of cancer, Wang et al. provided a revolutionary strategy, which can achieve nanoengineered EVs active targeting and controlled drug release: using RGD and FA as double ligand modification, combined gold nanorods (AuNRs) to selectively eliminate tumor cells under the irradiation of near-infrared light without affecting the surrounding normal tissues [151]. An emerging technique for EVs includes loading protein into the cytoplasm of target cells through EXPLORE technology (Fig. 7). Yim et al. developed an emerging technique for loading protein into the cytoplasm of target cells via light-inducible reversible protein–protein interactions (PPI). HEK293T cells were exposed to continuous blue light illumination, and the isolated EXPLORs contained significantly higher content of mCherry-cryptochrome 2(mCherry-CRY2) protein. In this system, a truncated version of CRY-interacting basic-helix-loop-helix 1 (CIBN) binds to CD9, cargo protein binds to CRY2, and the cargo protein was guided into the cell membrane surface under blue light irradiation. It is easy to separate the EVs carrying cargo protein from the cell. The subsequent series of experiments proved that using EXPLOR-based therapeutics can more effectively deliver proteins and transcription factors [152].

### Sonodynamic therapy

Ultrasound can enhance cell membrane permeability and promote the uptake of drugs by cancer cells and is commonly employed as an auxiliary tool for tumor treatment [153]. Liu et al. designed an advanced EVs-based drug delivery system: sinoporphyrin sodium as a sonosensitizer was encapsulated in EV and accumulated in large quantities at the tumor site. Under ultrasound stimulation, sinoporphyrin sodium is continuously released at the desired locations and generates reactive oxygen species (ROS) for therapeutic and imaging effects. As mentioned above, EVs had the highest uptake rate in homologous tumors, which was 2.35 times higher than that of other tumor cell lines. The in vivo fluorescence signal demonstrated the targeting of EVs to the tissues under the action of ultrasound, which ultimately exhibited excellent anti-tumor effects [154]. Similarly, Wang et al. demonstrated the significant role of ultrasound in EVs-targeted tumor therapy. They designed nanoengineered EVs carrying a payload to target tumor tissues with the assistance of ultrasound, improving the tumor immune microenvironment and working together with the EPR effect to inhibit tumor growth [100].

## Challenges and outlook

### Quantifying targeting efficiency

Current studies of EVs-targeted therapies typically use EVs as nanocarriers loading proteins or therapeutic RNAs that bind specifically to target cells, but little attention has been paid to the evaluation of various nanoengineered EVs-targeted therapeutic approaches. Commonly used methods for observing the distribution patterns of EVs include lipophilic fluorescently labeled EVs, X-ray, and positron emission tomography (PET) [155, 156]. Fluorescence imaging-based EVs monitoring is the most commonly used method for EVs tracking, which shows the accumulation of EVs modification groups over controls, but the technique is not very sensitive and does not provide absolute quantitative results. Therefore, a gold standard for quantifying the efficiency of targeted therapies is lacking.

### Adequate dosage of targeted EVs

An ongoing clinical trial for Alzheimer's disease has shown that MSCs-Exos are nasally dropped in amounts of 5–20 μg and is required twice a week for 12 weeks. These exosomes take several weeks to extract (ClinicalTrials.gov Identifier: NCT04388982). The selection of a suitable source of EVs for the experimental purpose was mentioned above as an effective way to address the low EV production. There is no clear method for meeting the volume of EVs required for large-scale clinical trials, and every process for producing therapeutically targeted EVs requires a safe protocol that reduces batch-to-batch variability. Alternatively, EVs mimics that are structurally and functionally similar to EVs but with higher yields are gaining interest in the field of cancer therapy [157]. Heterologous EV hybrids obtained by hybridizing macrophage-derived small cell EVs with synthetic liposomes using extruded membrane fusion techniques to facilitate the specific delivery of oncology drugs [158]. The integration of proteins associated with homing action in EVs mimics successfully confers corresponding tumor homing properties and can be used as an alternative to EVs [159]. Additionally, this approach is simpler, more convenient and high yielding, making it a promising method for targeted delivery of biomolecules.

### Environmental effects on EVs targeting

The environment to which EVs are exposed (pH and temperature) can significantly affect their uptake by the cells. Although low pH decreases the stability of EVs to some extent, it increases the uptake of EVs [160]. It has also been shown that an acidic environment alters the lipid composition of EVs and results in EVs possessing a more abundant negative charge on their surface, which allows EVs to better integrate with target cells [161]. It has been shown that the particle size of EVs increases as the temperature decreases. Compared to the freshly isolated EVs, the particle size of EVs increased by 10% and 25% at 4 °C and − 80 °C, respectively, however, the size of EVs decreased at 37 °C. Further characterization of the contents of the EVs at different temperatures showed that the decrease in temperature resulted in the leakage of some proteins [162]. Ge et al. demonstrated that the stability of miRNA at low temperatures was only slightly affected [163]. EVs are taken up more at 37 °C and 60 °C compared to 4 °C [160].

### Purity of EVs affects targeting

Although many studies have demonstrated that EVs are promising therapeutic vehicles for targeted drug delivery, the EVs that are currently isolated often contain "impurities" such as apoptotic vesicles or microvesicles [8]. Most people believe that the different biological components of vesicles affect the accuracy and reliability of EV-targeted therapies and that the quality and purity of EVs should be strictly required [164]. Therefore, the method of EV isolation may also be a factor affecting the distribution, as different isolation methods result in different protein/vesicle ratios [165]. The precise isolation of EVs from numerous vesicles has encountered critical challenges today. Current methods of EV isolation include the commonly used ultracentrifugation, gradient ultracentrifugation, and size-exclusion chromatography. Because to the lack of yield and purity of EVs, new enrichment methods have been developed recently that may better preserve the targeting function, such as microfluidic filtering, contact-free sorting, and immunoaffinity enrichment [166]. However, it has also been shown that these "impurities" in the EV extraction process can be involved in targeted therapies along with the EVs [164].

### Others

Some studies have also shown that the environment in which cells are cultured, such as hypoxia and low PH, can also enhance the EV production [167] and even enhance the targeting ability [10, 161, 168]. However, the composition of EV produced following cellular stress may be altered and the safety of EVs for therapeutic use at this time should be carefully assessed [160]. Additionally, in the EV-assisted targeting approach, the introduction of exogenous superparamagnetic nanoparticles to the surface of EVs does enhance the targeting properties [169], but it is unknown whether their biocompatibility is affected compared to that of the EVs themselves. Moreover, proteins, lipids, and glycans on the surface of EV membranes all play important roles in the targeting process, but most of the current research has focused on the surface modification of proteins [170]. Therefore, the modification methods for lipids and glycans should be further explored subsequently. Finally, it is uncertain whether the various modification methods we mentioned above for EVs will cause damage to the membrane proteins on their surface and thus affect the targeted delivery function of EVs. Therefore, subsequent experiments are needed to further study these issues and to develop safe and non-toxic strategies for the design of targeted EVs.

## Conclusions

EVs, as cell-released vesicles, are emerging as promising nanoplatforms for drug delivery. Natural EVs themselves abundantly express unique membrane surface molecules including proteins, lipids, and glycans that facilitate EV targeting mission. Moreover, EVs injection modalities and parent cells determine the fate of EVs after release into the extracellular matrix. In order to upgrade natural EV-targeted drug delivery capabilities, researchers explored a range of nanoengineering approaches, including surface display and post-isolation methods. We further compared different nanoengineered EVs strategies. Several non-invasive external stimuli can assist natural and nanoengineering EVs in targeted drug delivery. Aggregation of therapeutic EVs to the injured tissue by magnetism, ultrasound or photothermal can reduce off-target effects of EVs and avoid damage to normal tissues. Despite these encouraging achievements, there are still some obstacles to the application of EVs in precision delivery platforms, such as the lack of a gold standard for measuring EVs targeting efficiency and the need to overcome the problem of insufficient EVs yield. Thus, more advanced approaches are still needed to expand the potential of EVs in targeted therapies. In the future, we expect a multidisciplinary effort to achieve a scale-up production of quality-controlled targeted EVs and break the therapeutic bottleneck of EVs in targeted therapy.

## Data Availability

Data sharing is not applicable to this article as no datasets were generated or analyzed during the current study.

## Abbreviations

AA-PEG: Aminoethylanisamide-polyethylene glycol
ABP: Aptamer-binding protein
AnV: Annexin V
ASGPR: Asialoglycoprotein receptor
AuNRs: Gold nanorods
BBB: Blood–brain barrier
BMSC: Bone marrow mesenchymal stem cells
BSM: Bone-marrow derived macrophages
CAP: Chondrocyte-affinity peptide
CCL18: Chemokine (C–C motif) ligand 18
CCR8: Chemokine (C–C motif) receptor 8
CD47: Cluster of differentiation 47
CDCs: Cardiosphere-derived cells
CIBN: CRY-interacting basic-helix-loop-helix 1
CML: Chronic myeloid leukemia
CMP: Cardiomyocyte specific peptide
CPCs: Cardiac progenitor cells
Cur: Curcuminoid
CXCR4: C-X-C motif chemokine receptor 4
DC: Dendritic cell
DC-SIGN: Dendritic cell-specific ICAM-grabbing non-integrin
DMD: Duchenne muscular dystrophy
DMPE-PEG: Glycerol-phospholipid-PEG conjugates
Dox: Doxorubicin
EC: Endothelial cells
EGF: Epidermal growth factor
EGFR: Epidermal growth factor receptor
EpCAM: Epithelial cell adhesion molecule
EPR: Enhanced permeability and retention
EVs: Extracellular vesicles
EXO: Exosome
EXO_KV11_: EXO-linked KV11 peptide
FA: Folate
GAP43: Growth-associated protein-43
HCT-1165FR: 5-FU-resistant derivative of the HCT-116 human colon cancer cell line
HDEA: Hyaluronic acid grafted with 3-(diethylamino) propylamine
HEK293: Human embryonic kidney 293
HuR: Human antigen R
ICAM-1: Intercellular-adhesion molecule 1
ICDCs: Cardiosphere-derived cells
IL3: Interleukin 3
IL-4: Interleukin-4
imDC: Immature dendritic cells
IMTP: Ischemic myocardium-targeting peptide
IONP: Iron oxide nanoparticles
KV11: A known anti-angiogenic factor
LA: Lactadherin
Lamp-2b: Lysosome-associated membrane protein-2b
LFA-1: Lymphocyte function associated antigen 1
mCherry-CRY2: MCherry-cryptochrome 2
MFs: Magnetic fields
MHC: Major histocompatibility complex
MOR: Opioid receptor Mu
MSC: Mesenchymal stem cells
MUC1: Mucin 1(MUC1)
MVBs: Multi-vesicular bodies
NE: Neutrophil
NRP1: Neuropilin1
NRP2: Neuropilin2
OA: Osteoarthritis
OIR: Oxygen-induced retinopathy
PD: Parkinson disease
PDGFR: Platelet-derived growth factor receptor
PEG: Polyethylene glycol
PEI: Polyethyleneimine
PET: Positron emission tomography
PPI: Protein–protein interactions
PS: Phosphatidylserine
PSR: Phosphatidylserine receptor
PTX: Paclitaxel
Que: Quercetin
Raw264.7: Leukemia cells in mouse macrophage
RES: Reticuloendothelial system
RGD: Arg-Gly-Asp
RGE: Neuropilin-1-targeted peptide
ROS: Reactive oxygen species
RVG: Rabies viral glycoprotein
SD rat: Sprague–Dawley rat
SF-MSC: Synovial fluid-derived mesenchymal stem cells
SIRPα: Signal regulatory protein-α
SMCNCs: Superparamagnetic magnetite colloidal nanocrystal clusters
SPIONs: Superparamagnetic iron oxide nanoparticles
ST: Stromal cell
Tf: Transferrin
TfR: Transferrin receptors
TIM4: T-cell immunoglobulin mucin 4
TRAIL: TNF-related apoptosis-inducing ligand
Tstan8: Tetraspanin-8
USPIO: Ultrasmall superparamagnetic iron oxide
